# Role of endogenous and exogenous antioxidants in risk of six cancers: evidence from the Mendelian randomization study

**DOI:** 10.3389/fphar.2023.1185850

**Published:** 2023-06-27

**Authors:** Jiahao Zhu, Jie Lian, Xin Wang, Ren Wang, Xiangyi Pang, Benjie Xu, Xing Wang, Chenyang Li, Shengjun Ji, Haibo Lu

**Affiliations:** ^1^ Harbin Medical University Cancer Hospital, Department of Outpatient Chemotherapy, Harbin, China; ^2^ The Affiliated Suzhou Hospital of Nanjing Medical University, Department of Radiotherapy and Oncology, Suzhou, China

**Keywords:** oxidative stress, endogenous antioxidant, exogenous antioxidant, cancer prevention, Mendelian randomization study

## Abstract

**Background:** Although oxidative stress is known to contribute to cancer, and endogenous and exogenous antioxidants are thought to prevent tumorigenesis by suppressing oxidative stress-induced DNA damage, antioxidants have also been reported to show negative effects on tumor formation, necessitating characterization of the causal associations between antioxidants and cancer risk.

**Methods:** In this study, Mendelian randomization (MR) analysis, primarily inverse-variance weighted MR, was used to assess the causal effect of six endogenous and five exogenous diet-derived antioxidants on the risk of six cancers. MR-Egger intercept test and Cochran’s Q statistic were utilized to assess pleiotropy and heterogeneity, respectively.

**Results:** For endogenous antioxidants, a bidirectional two-sample MR analysis was conducted. Our findings suggested that serum albumin has a negative causal association with the risk of prostate cancer [odds ratio (OR) = 0.78, 95% confidence interval (CI): 0.68–0.91, *p* = 0.001]. The risks of the six cancers showed no significant associations with endogenous antioxidants in the converse MR analysis. For exogenous antioxidants, the unidirectional two-sample MR analysis exhibited a nominal relationship between the serum retinol level and non-small-cell lung cancer risk (OR = 0.29, 95% CI: 0.11–0.76, *p* = 0.011).

**Conclusions:** Thus, our study revealed the protective effects of genetic susceptibility to high circulating albumin levels on prostate cancer, providing potential targeted interventions for prostate cancer prevention.

## Introduction

Cancer is a major cause of death worldwide, with about 10 million deaths reported in 2020 (Sung et al., 2021). According to the latest global cancer statistics, female breast cancer has surpassed lung cancer as the most commonly diagnosed cancer; however, lung cancer remains the paramount cause of cancer mortality. Lifestyle changes in the developed regions of the world and the absence of effective preventive screening programs and healthcare access in some less-developed regions are expected to further increase cancer-related morbidity and mortality in the future (Sung et al., 2021).

**FIGURE 1:**
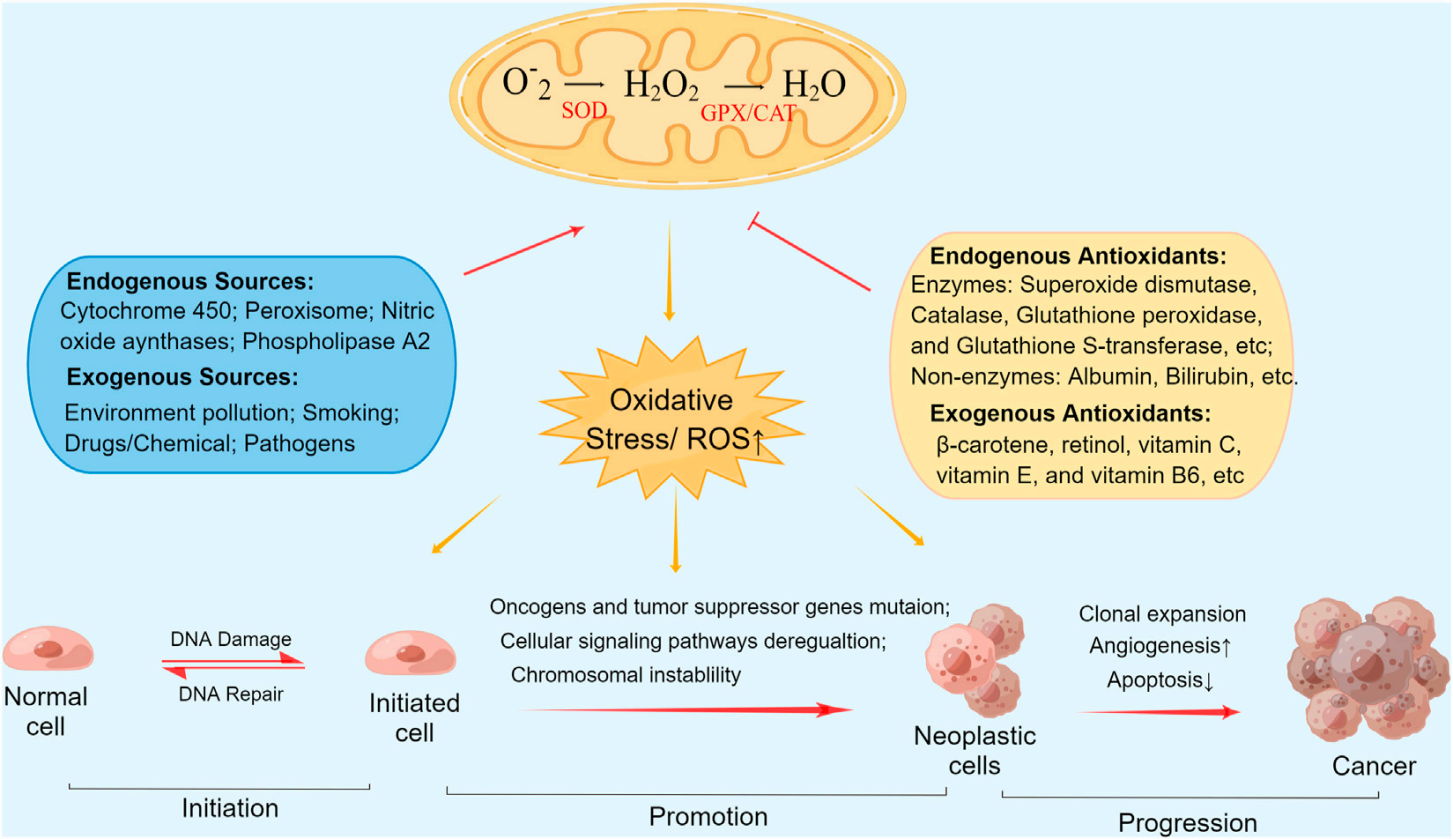
Antioxidants actions in tumorigenesis and mechanistic pathways. Abbreviations: ROS, Reactive oxygen species; SOD, superoxide dismutase; CAT, catalase; GPX, glutathione peroxidase.

The causal and contributory role of oxidative stress in tumorigenesis has been described previously (Klaunig, 2018). While the association between reactive oxygen species (ROS) and antioxidants in humans is usually well balanced to maintain normal metabolic activity, excessive ROS generation or decreased antioxidative activity resulting from any cause may lead to oxidative stress and thereby induce adverse effects and diseases, including cancer (Milkovic et al., 2014). Different concentrations of ROS contribute to various levels of damage to biological macromolecules (Trachootham et al., 2008). Excess ROS, if not detoxified by antioxidants, can lead to irreversible genomic damage, lipid peroxidation, persistent mitochondrial dysfunction, and activation of aberrant signaling pathways, resulting in cellular mutation, chromosome instability, and neoplasm initiation (Milkovic et al., 2014). Endogenous and exogenous antioxidant systems maintain cellular redox homeostasis by scavenging ROS through various mechanisms (Harris and DeNicola, 2020). Figure 1 depicts antioxidants actions in tumorigenesis and mechanistic pathways. Endogenous antioxidants consist primarily of albumin, bilirubin, and enzymes involved in antioxidation, including superoxide dismutase (SOD), catalase (CAT), glutathione peroxidase (GPX), and glutathione S-transferase (GST). Principal exogenous antioxidants, namely, diet-derived antioxidants, include β-carotene, retinol, vitamin C, vitamin E, and vitamin B6 (Nordberg and Arnér, 2001; Parra et al., 2018).

Antioxidants can theoretically prevent tumorigenesis, and their cancer-suppressing function has been observed in several mouse tumor models. Multiple molecular subtypes of GST have shown the ability to prevent skin, colon, and liver cancer initiation in mice exposed to carcinogens or lacking tumor suppressors (Abel et al., 2010; Henderson et al., 2011; Li et al., 2019). Barrett et al. demonstrated that GPX3-deficient mice were vulnerable to various tumors with poor differentiation, while GPX3 suppressed the progression of colitis-associated carcinoma through immunomodulatory effects and by inhibiting the oxidative stress response (Barrett et al., 2013). Additionally, a higher incidence of cancer and DNA damage was observed in mice deficient in SOD1 (Van Remmen et al., 2003). However, endogenous antioxidants also play a paradoxical role in tumor development. One recent study reported that depletion of glutathione (GSH) contributes to protein homeostasis and cell survival in tumors through deubiquitinating enzymes (Harris et al., 2019). GST and GPX enzymes participating in the downstream pathway of GSH utilization have also been implicated in tumor development (Harris and DeNicola, 2020). In terms of exogenous antioxidants, two aspects have been shown to be involved in cancer initiation. The Iowa Women’s Health Study found that the incidence of colorectal cancer was lower in populations with a high intake of vitamin E, especially among older individuals (Bostick et al., 1993). Similarly, the outcome of the ATBC trial also confirmed a significant negative relationship between α-tocopherol levels and risk of prostate cancer. The α-tocopherol group also showed lower prostate cancer-related mortality at long-term follow-up [15]. However, a diametrically opposite result was obtained in another study. The SELECT trial to investigate the preventive role of vitamin E in prostate cancer (Lippman et al., 2005; Lippman et al., 2009; Klein et al., 2011) was terminated prematurely because of the higher incidence of prostate cancer in the experimental group. Additionally, vitamin E, A, and C intake were not associated with breast cancer risk (Kushi et al., 1996). Thus, the actual influence of these antioxidants in cancer prevention is currently unclear.

Potential confounding factors, limited sample sizes, and other biases in observational studies make it difficult to derive an undetermined causal conclusion between antioxidants and cancers. In this regard, Mendelian randomization (MR) can serve as an effective analytical approach to identify the role of antioxidants in cancer. In MR, genetic variants are applied as instrumental variables (IVs) to access the potential causal relationship between exposure and outcome due to the random distribution of these variants during meiosis (Davey Smith and Hemani, 2014). The germline genotype is formed before the onset and progression of the disease, which further minimizes the possibility of confounding. In our study, a bidirectional two-sample MR was used to assess whether endogenous antioxidants were related to six cancers, and a unidirectional two-sample MR was performed to access the causal relationship between exogenous antioxidants and the six cancers.

## Materials and methods

### Study design

**FIGURE 2:**
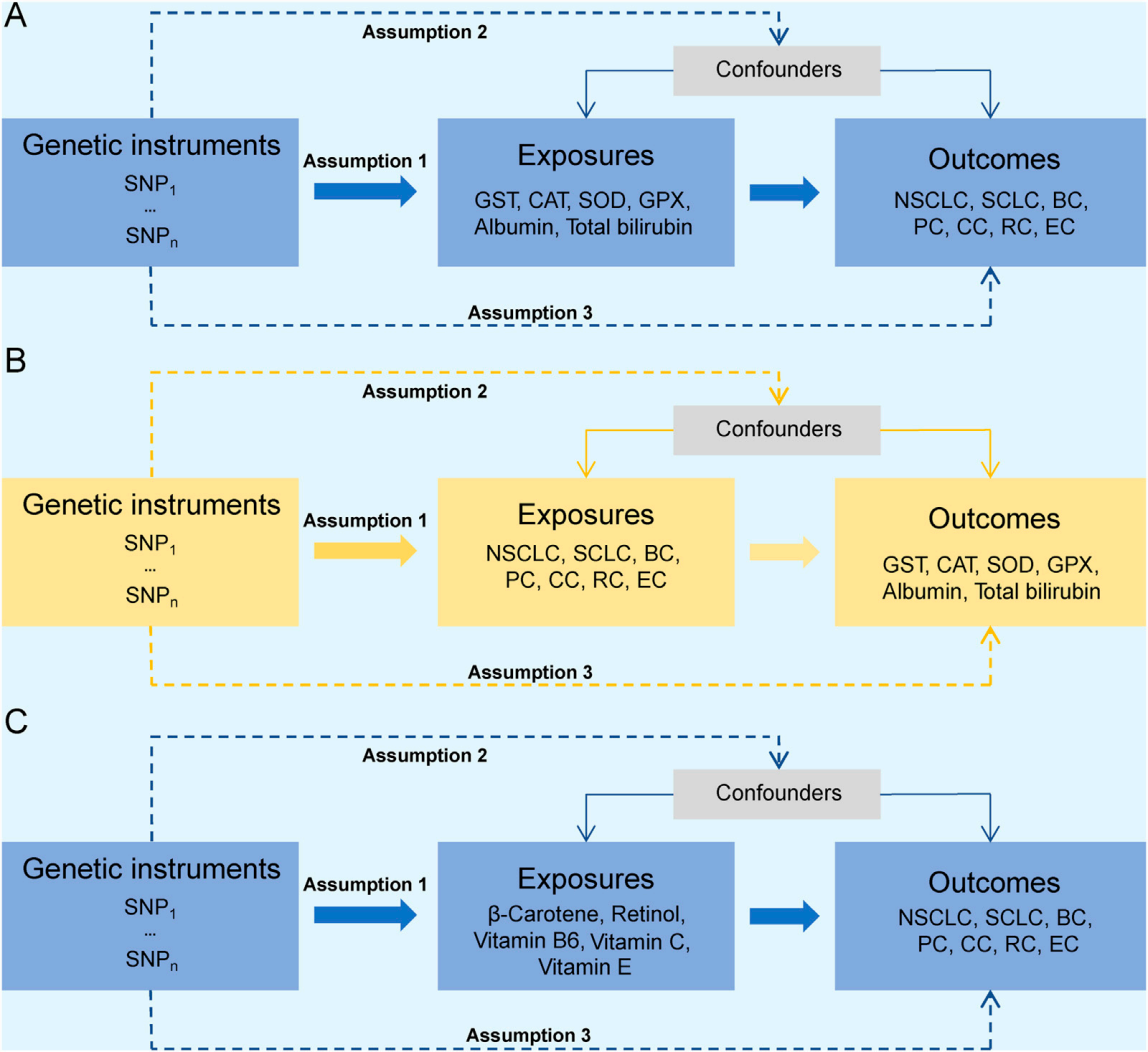
Study design overview. **(A, B)** Flow chart of the bidirectional MR study design between endogenous antioxidants and cancer risks. **(C)** Flow chart of the unidirectional MR study design between exogenous antioxidants and cancer risks. Abbreviations: SNP, single-nucleotide polymorphism; MR, Mendelian randomization; GST, glutathione s-transferase; SOD, superoxide dismutase; CAT, catalase; GPX, glutathione peroxidase; NSCLC, non-small-cell lung cancer; SCLC, small-cell lung cancer; BC, breast cancer; PC, prostate cancer; CC, colon cancer; RC, rectum cancer; EC, esophageal cancer.

An overview of the bidirectional and unidirectional two-sample MR designs is presented in Figure 2. Bidirectional MR was conducted to access the causal association between the six endogenous antioxidants and the six cancers (Figures 2A, B). Unidirectional MR was performed to assess the causal association between five exogenous antioxidants and the six cancers (Figure 2C). The endogenous antioxidants evaluated in this study included GST, CAT, SOD, GPX, albumin, and total bilirubin. The exogenous antioxidants included β-carotene, retinol, and vitamins B6, C, and E. Given that our research primarily centers on the incidence of tumors, four tumors with the highest incidence rate were included and analyzed, including breast (morbidity: 11.7% of total cases; mortality: 6.9%), followed by lung (morbidity: 11.4%; mortality: 18.0%), colorectal (morbidity: 10.0%; mortality: 9.4%), and prostate (morbidity: 7.3%; mortality: 3.8%) cancers (Sung et al., 2021). Due to the biological and pathological difference, lung cancer was divided into non-small cell lung cancer and small cell lung cancer for analysis. Esophageal cancer, which has a relatively low incidence rate (3.1%) but high mortality rate (5.5%), was also included in the analysis. After an integrated consideration of all cancer incidence and mortality rates, the following six cancers were selected for further analysis: non-small-cell lung cancer (NSCLC), small-cell lung cancer (SCLC), breast cancer (BC), prostate cancer (PC), colon cancer (CC), rectal cancer (RC), and esophageal cancer (EC). The main assumptions for MR were as follows: (1) the genetic predictors from genome-wide association studies (GWAS) served as instrumental variables (IVs) that are strongly associated with the exposure phenotypes; (2) the genetic predictors are independent of confounders; and (3) the genetic predictors are associated with the outcome only by affecting the exposure, with no other pathways (Lawlor et al., 2008).

### Associations of genetic IVs with endogenous and exogenous antioxidants

IVs of the six endogenous and five exogenous antioxidants were extracted from the most recent GWAS. Genetic variants of the six endogenous and five exogenous antioxidants were extracted from the most recent GWAS. Data for GST, CAT, SOD, and GPX were obtained from the INTERVAL study (Sun et al., 2018). Albumin data were obtained from the KORA study (Shin et al., 2014). Data for total bilirubin and all five exogenous antioxidants, namely, β-carotene, retinol, and vitamins B6, C, and E, were obtained from the UK biobank (Kimberley and Philip, 2021; Ruth et al., 2019). The number of samples was as follows: GST, 3301 cases; CAT, 3301 cases; SOD, 3301 cases; GPX, 3301 cases; albumin, 115060 cases; total bilirubin, 342829 cases; β-carotene, 64979 individuals; retinol, 62991 individuals; vitamin B6, 64979 individuals; vitamin C, 64979 individuals; vitamin E, 64979 individuals. All participants were Europeans. The detailed characteristics of these 11 antioxidants are shown in Table 1.

**TABLE 1:** Detailed information regarding studies and datasets used in the present study.

Exposures or outcomes	Traits	Abbreviation	Dataset	Ancestry	Participants (cases/controls)	Total *R* ^2^ (exposures)	F-statistics (exposures)	Web source (accessed on 10 December 2022)
Endogenous antioxidants	Glutathione S-transferase	GST	INTERVAL study (PMID:29875488)	European	3301	0.2229	52.2968	https://gwas.mrcieu.ac.uk/datasets/prot-a-1283/
	Catalase	CAT	INTERVAL study (PMID:29875488)	European	3301	0.1317	31.1421	https://gwas.mrcieu.ac.uk/datasets/prot-a-367/
	Superoxide dismutase	SOD	INTERVAL study (PMID:29875488)	European	3301	0.0993	27.8713	https://gwas.mrcieu.ac.uk/datasets/prot-a-2800/
	Glutathione peroxidase	GPX	INTERVAL study (PMID:29875488)	European	3301	0.2950	54.8247	https://gwas.mrcieu.ac.uk/datasets/prot-a-1265/
	Albumin	—	KORA study (PMID:24816252)	European	115060	0.0504	44.5391	https://gwas.mrcieu.ac.uk/datasets/met-d-Albumin/
	Total bilirubin	—	United Kingdom Biobank	European	342829	0.5020	657.2863	https://gwas.mrcieu.ac.uk/datasets/ukb-d-30840_raw/
Exogenous antioxidants	β-Carotene	—	United Kingdom Biobank	European	64979	0.0055	22.6428	https://gwas.mrcieu.ac.uk/datasets/ukb-b-16202/
	Retinol	—	United Kingdom Biobank	European	62991	0.0029	23.2573	https://gwas.mrcieu.ac.uk/datasets/ukb-b-17406/
	Vitamin B6	—	United Kingdom Biobank	European	64979	0.0061	23.3452	https://gwas.mrcieu.ac.uk/datasets/ukb-b-7864/
	Vitamin C	—	United Kingdom Biobank	European	64979	0.0039	23.1618	https://gwas.mrcieu.ac.uk/datasets/ukb-b-19390/
	Vitamin E	—	United Kingdom Biobank	European	64979	0.0043	23.1775	https://gwas.mrcieu.ac.uk/datasets/ukb-b-6888/
Cancers	Non-small-cell lung cancer	NSCLC	FinnGen Biobank	European	1627/174006	0.0022	27.6700	https://gwas.mrcieu.ac.uk/datasets/finn-b-C3_LUNG_NONSMALL_EXALLC/
	Small-cell lung cancer	SCLC	FinnGen Biobank	European	179/174006	0.0013	22.5473	https://gwas.mrcieu.ac.uk/datasets/finn-b-C3_SCLC_EXALLC/
	Breast cancer	BC	FinnGen Biobank	European	8401/99321	0.0232	33.2302	https://gwas.mrcieu.ac.uk/datasets/finn-b-C3_BREAST_EXALLC/
	Prostate cancer	PC	FinnGen Biobank	European	6311/74685	0.0689	45.6863	https://gwas.mrcieu.ac.uk/datasets/finn-b-C3_PROSTATE_EXALLC/
	Colon cancer	CC	FinnGen Biobank	European	1803/174006	0.0016	22.9942	https://gwas.mrcieu.ac.uk/datasets/finn-b-C3_COLON_EXALLC/
	Rectum cacner	RC	FinnGen Biobank	European	1078/174006	0.0019	23.2223	https://gwas.mrcieu.ac.uk/datasets/finn-b-C3_RECTUM_EXALLC/
	Esophagus cancer	EC	FinnGen Biobank	European	232/174006	0.0012	22.7477	https://gwas.mrcieu.ac.uk/datasets/finn-b-C3_OESOPHAGUS_EXALLC/

### Associations of genetic IVs with the six cancers

The GWAS data of the six cancers, including NSCLC (1627 cases and 174006 controls), SCLC (179 cases and 174006 controls), BC (8401 cases and 99321 controls), PC (6311 cases and 74685 controls), CC (1803 cases and 174006 controls), RC (1078 cases and 174006 controls), and EC (232 cases and 174006 controls), were obtained from the FinnGen (https://www.finngen.fi/en) study of European individuals. The GWAS summary statistics in the FinnGen study are available in the IEU OpenGWAS database (Hemani et al., 2018). Detailed characteristics of the six cancers are shown in Table 1.

### Selection of genetic instrumental variables

Single-nucleotide polymorphisms (SNPs) that were strongly and independently (*R* ^2^ < 0.1) associated with exposure and distance <10 KB was selected at a genome-wide significance level of *p* < 5 × 10^−6^. SNP-specific F-statistics were calculated to evaluate the instrumental efficiency of SNPs with *R* ^2^, exposure sample size, and the number of SNPs (Bowden et al., 2016). SNPs were deleted if their F-statistic was <10. Exposure to fewer than three independent SNPs was omitted. We scanned the GWAS catalog to reveal the associations between SNPs and other potential confounders to reduce the impact of pleiotropic IVs on MR results (Supplementary Table S1) (Buniello et al., 2019).

### Statistical analysis

An inverse-variance weighted (IVW) analytical approach was applied to provide MR estimation and showed the largest statistical power among all MR methods by combining each Wald ratio of multiple SNPs (Burgess et al., 2017). The slope and intercept of the MR-Egger plot indicated the potential causal estimates and degree of pleiotropy, respectively (Bowden et al., 2015). The MR-Egger method not only allows for testing and estimation of causal effects similar to the IVW method, but also facilitates evaluation of directional pleiotropy through the addition of a parameter, denoted as 𝜃_0_. In contrast to the IVW approach which sets the intercept term to zero, the introduction of the aforementioned parameter in the MR-Egger framework enables assessment of pleiotropic effects that may be distributed independently from those of instrumental variables (IVs) on outcomes through exposures. Notably, non-fulfillment of Assumption 3 in Figure 2 ensues when the value of 𝜃_0_ deviates from zero, thereby rendering the MR-Egger estimate different from that of the IVW method. To this end, the pleiotropy test entails determining whether the said intercept term is precisely equal to zero. Weighted median is another MR method to calculate the causal estimate by combining data of multiple genetic IVs. A consistent estimation of the causal effect can be obtained using the weighted median method if the weighted median of the SNP-specific estimates reaches 50% (Bowden et al., 2016). In comparison to the IVW method, the weighted median approach exhibits superior Type 1 error rates in finite samples, and is also regarded as a valuable complement to the MR-Egger method. Potential outliers can be detected by Mendelian Randomization Pleiotropy RESidual Sum and Outlier (MR-PRESSO) analysis, and causal estimates can be obtained after removing outlier variants (Verbanck et al., 2018). Additionally, heterogeneity among the SNPs was assessed using Cochrane’s Q test. The Bonferroni method was used for multiple comparisons, and “leave-one-out” analyses were conducted to evaluate the stability of our findings. We defined *p*-values below 0.0012 (0.05/42) as indicating strong evidence of associations in the MR analysis between the six endogenous antioxidants and six cancers. *p*-values <0.0014 (0.05/35) indicated strong evidence of relationships between the five exogenous antioxidants and six cancers. Suggestive associations were considered if *p*-values were between 0.05 and the respective values indicating strong associations. The strength of the instrumental variables (IVs) was assessed using the F-statistic. This statistical measure allows for an estimation of the minimum detectable magnitude of a causal association in MR. Detailed information regarding the other methods applied in this study is provided in the Supplementary Material. The results of the effects of the 11 antioxidants on cancers are presented as odds ratios (ORs) (95% confidence intervals (CIs)) per 1 standard deviation (SD) genetic predicted antioxidant change, and the effects of the cancers on the six endogenous antioxidants were reported as β coefficients and 95% CIs. All analyses were two-sided and performed using R software (Version 4.0.2) with the “TwosampleMR” and “MR-PRESSO” R packages.

## Results

### Characteristics of the instrumental variables

All 11 antioxidants and six cancers had three or more independent genome-wide significant SNPs. Supplementary Tables S2–S5 shows the final SNPs included in further MR analyses. The characteristics of each exposure and genetic IVs are shown in Table 1. The F-statistics of MR ranged from 22.54 to 657.29, suggesting that weak instrumental bias may not have been substantial in our study (Palmer et al., 2012). No directional pleiotropy was observed in these filtered IVs, implying that the MR assumptions were unlikely to be violated.

### Causal effect of endogenous antioxidants on the risk of six cancers

**FIGURE 3:**
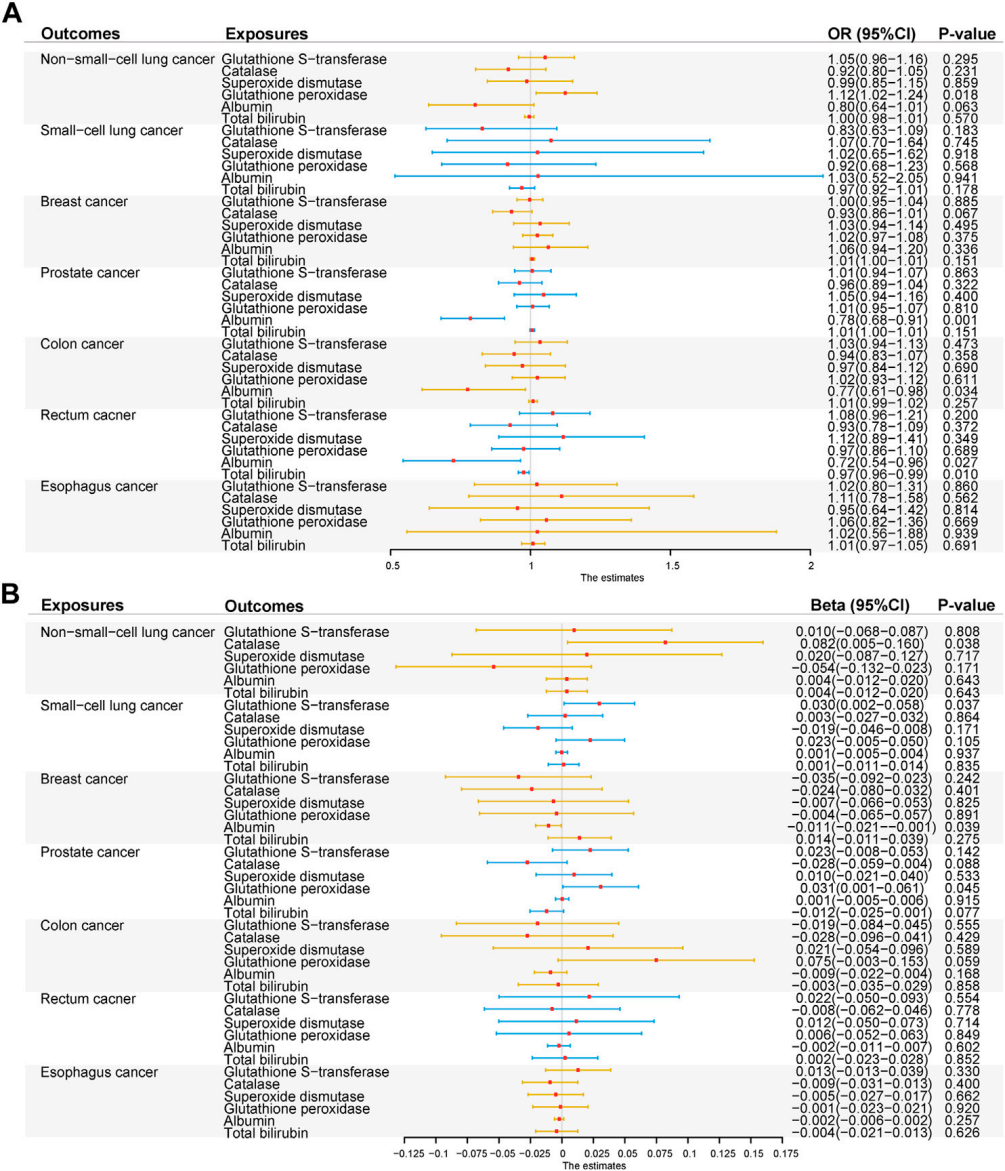
Summary of the inverse-variance weighted (IVW) results. **(A)** IVW results of forward-direction Mendelian randomization analysis between six genetically predicted circulating endogenous antioxidants and six cancers. **(B)** IVW results of reverse-direction Mendelian randomization analysis between six genetically predicted circulating endogenous antioxidants and six cancers. Abbreviations: GST, glutathione s-transferase; CAT, catalase; SOD, superoxide dismutase; GPX, glutathione peroxidase.

**FIGURE 4:**
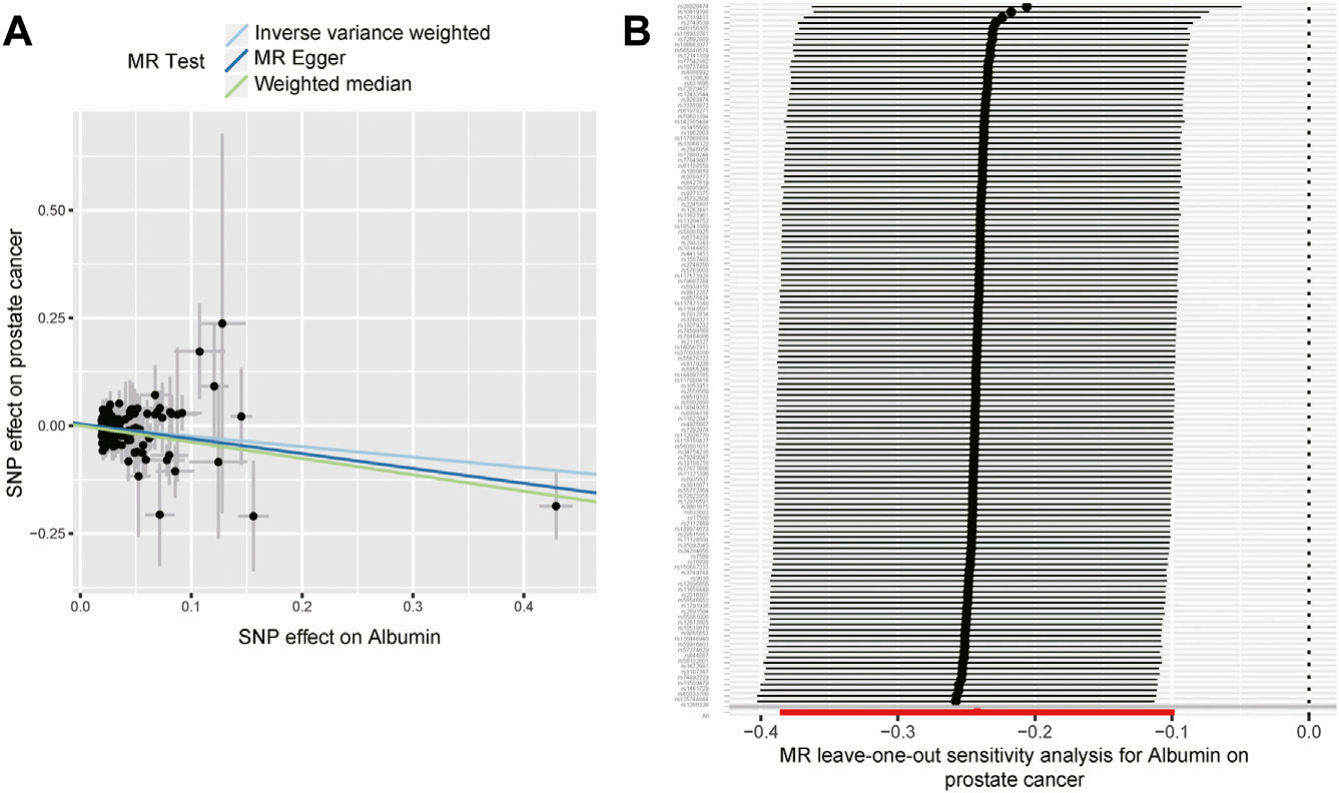
The effect of circulating albumin on the risk of prostate cancer. **(A)** The effect estimators of circulating albumin on the risk of prostate cancer based on three Mendelian randomization methods (inverse-variance weighted, MR-Egger, and weighted median method). **(B)** Leave-one-out sensitivity analysis for serum albumin on prostate cancer.

The MR association estimates of the causal association between six endogenous antioxidants and the risks of six cancers are shown in Figure 3A, and detailed outcomes are shown in Supplementary Table S5. IVW showed that a higher level of serum albumin was strongly associated with a decreased risk of PC (OR = 0.78; 95% CI: 0.68–0.91, *p* = 0.001). The same effect of serum albumin on PC was also observed in the MR-Egger method (OR = 0.71; 95% CI: 0.54–0.93; *p* = 0.013) and weighted median (WM) analyses (OR = 0.68; 95% CI: 0.53–0.88; *p* = 0.003). No horizontal pleiotropy was detected in the MR-PRESSO global test or the MR-Egger intercept test. No heterogeneity was observed in the Cochran’s Q test. Leave-one-out sensitivity analysis showed the stability of serum albumin genetic prediction efficiency (Figure 4). Nominal associations between endogenous antioxidants and cancer also existed. A higher level of GPX was associated with increased NSCLC risk (OR = 1.12; 95% CI: 1.02–1.24; *p* = 0.018). Circulating albumin levels had negative nominal associations with CC risk (OR = 0.77; 95% CI: 0.61–0.98; *p* = 0.034) and RC risk (OR = 0.72; 95% CI: 0.54–0.96; *p* = 0.028). Suggestive associations also existed between total bilirubin and RC risk (OR = 0.97; 95% CI: 0.96–0.99; *p* = 0.010).

### Causal effect of six cancers on endogenous antioxidants

Based on the Bonferroni-corrected threshold, no significant causal association between the six cancers and endogenous antioxidants was found in the reverse MR analysis (Figure 3B); the detailed outcomes are shown in Supplementary Table S6. NSCLC was found to show a suggestive relationship with higher CAT levels (*β* = 0.08; 95% CI: 0.00–0.16; *p* = 0.038). A positive nominal association between SCLC and GST level was also observed (*β* = 0.03; 95% CI: 0.00–0.06; *p* = 0.037). Suggestive associations also existed between BC and decreased levels of albumin (*β* = −0.01; 95% CI: -0.02 to 0.00; *p* = 0.039) and between PC and increased levels of GPX (*β* = 0.03; 95% CI: 0.00–0.06; *p* = 0.045).

### Causal effect of exogenous antioxidants on the risk of the six cancers

**FIGURE 5:**
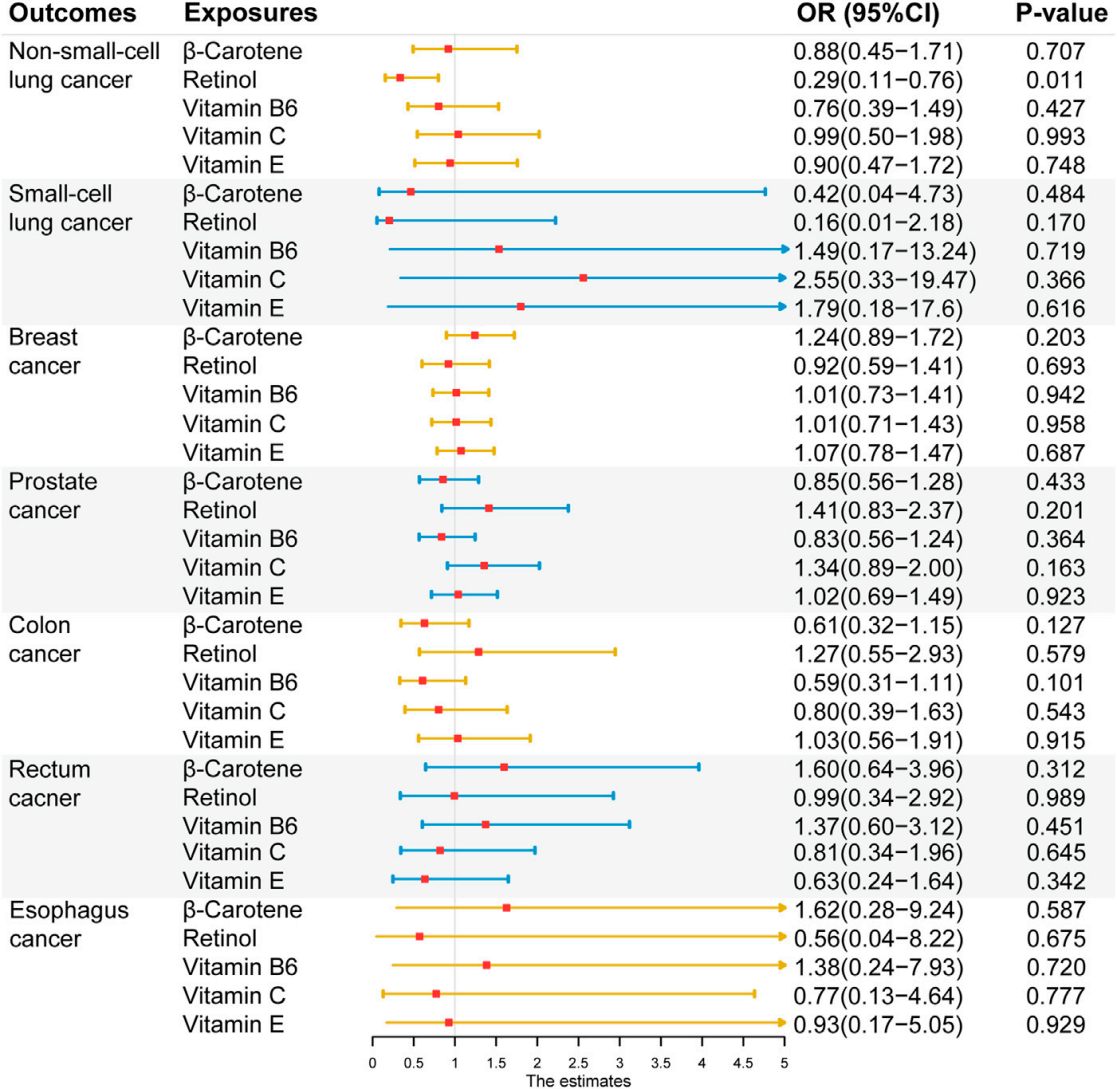
Associations between genetically five predicted circulating exogenous antioxidants and six cancer risks (odds ratio (OR) and 95% confidence interval (CI) represent the change in OR of cancers per 1 standard deviation increase in each exogenous antioxidants in the blood).

After Bonferroni correction, no significant causal association was found between exogenous antioxidants and the six cancers (Figure 5). Detailed outcomes are shown in Supplementary Table S7. Suggestive evidence was observed between the level of retinol and NSCLC risk (IVW method: OR = 0.29; 95% CI: 0.11–0.75; *p* = 0.011; MR-Egger method: OR = 0.51; 95% CI: 0.06–4.22; *p* = 0.561 and weighted median (WM) method: OR = 0.30; 95% CI: 0.09–0.98; *p* = 0.045).

## Discussion

In this study, bidirectional and unidirectional MR analyses were conducted to assess the causal relationship between endogenous and exogenous antioxidants and the six types of cancer. The outcomes indicated that a higher level of serum albumin had a significant causal association with a decreased risk of PC. No other strong relationships were observed in our findings. Meanwhile, nominal evidence was observed for a possible causal effect of GPX on increased NSCLC risk, albumin on the risk of CC and RC, and total bilirubin on RC risk. Constitutive causal effects of the six cancers on endogenous antioxidants were also detected: NSCLC was suggestively associated with increased CAT levels; SCLC showed a suggestive association with increased GST levels; BC was nominally correlated with decreased albumin levels; PC showed a positive causal relationship with GPX; and retinol was the only exogenous antioxidant showing a causal association with NSCLC.

The involvement of oxidative stress in numerous diseases, including tumorigenesis, has been widely acknowledged (Thyagarajan and Sahu, 2018). Mitochondria are the primary organelles that produce ROS and free radicals and reduce these products of metabolism; however, their clearance function *in vivo* is limited. Therefore, antioxidants are, in theory, crucial for maintaining body homeostasis and decreasing cancer risk. The assumption of the antioxidative and anti-inflammatory properties of albumin may remain biologically plausible and circulating albumin has also been observed to have an inverse association with the risk of cardiovascular disease morbidity and mortality (Danesh et al., 1998; Putin et al., 2016). In this study, higher serum albumin levels showed a significant causal relationship with PC risk, but no significant association was observed in reverse-causality analysis. Although several previous studies have reported no significant relationship between the albumin level and risk of PC, a weaker inverse association has been reported (Van Hemelrijck et al., 2011; Kühn et al., 2017). Limited numbers of samples, multiple potential confounders, and uncertain causality may have masked the actual association between albumin concentration and PC risk. MR can rule out residual confounding in observational studies and provide a more plausible estimation of causation. To our knowledge, this is the first robust causal evidence for the potential preventive effects of serum albumin on PC development. Albumin was also found to be an independent overall survival prognosis indicator in castration-resistant PC (Chi et al., 2016). It seems that albumin may play a significant role in the initiation and progression of PC. Additionally, our study also showed a suggestive inverse association between the level of albumin and CC risk, in line with the weak inverse associations observed in two previous studies (Ghuman et al., 2017; Kühn et al., 2017). Overall, these previous outcomes and the suggestive inverse associations reported in this study may again point to a weak inverse relationship between albumin level and the risk of RC. Result of early study also indicated a negative association between albumin levels and CC risk (Ko et al., 1994). The lack of a relationship between albumin levels and lung cancer risk in this study is consistent with the reports from two prospective studies based on American and European cohorts (Sprague et al., 2008; Kühn et al., 2017). No significant causal association between albumin concentrations and the risk of BC was found in our study, which is consistent with the findings of the Swedish AMORIS study (Wulaningsih et al., 2015). Interestingly, we observed that BC had a suggestive causal relationship with decreased albumin levels, which may account for the negative association between albumin and BC risk observed in the EPIC study (Kühn et al., 2017). In terms of endogenous enzymatic antioxidants, no significant causal correlation was found in both forward- or reverse-direction MR analyses. Endogenous enzymatic antioxidants can prevent oxidative DNA lesions induced by ROS and other free radicals with a high efficiency. However, their direct role in preventing tumorigenesis resulting from oxidative stress remains uncertain because of the application of carcinogen-induced tumor models and the inevitable carcinogen detoxification by antioxidants (Harris and DeNicola, 2020). The pro-tumor or antitumor effects of endogenous antioxidants have been observed in various cancers and even in the same cancer (Harris and DeNicola, 2020). Further studies are warranted to explore the potentially beneficial pathways.

In assessments of exogenous antioxidants, only an inverse nominal association between retinol level and NSCLC risk was observed in this study. And no significant horizontal pleiotropy and heterogeneity was detected based on the outcomes of MR-Egger outcomes. The biological function of retinol has long been thought to attributable to retinoic acid. However, a recent study demonstrated the anticancer effects of retinol (Li et al., 2016). Retinol suppressed the adhesion of PC cells and inhibited PC cell proliferation in a dose-dependent manner, indicating that it could inhibit PC progression and metastasis. Nevertheless, few studies to date have investigated the antitumor effects of retinol on lung cancer and other malignancies. β-carotene, a precursor of vitamin A, exerts antioxidant effects by scavenging singlet oxygen (Takahashi et al., 2022). Previous observational studies have reported an inverse association between β-carotene levels and several cancers, including breast, lung, liver, and pancreatic cancers (Bakker et al., 2016; Huang et al., 2016; Kataria et al., 2016; Shareck et al., 2017). However, no significant causal relationship was observed between β-carotene and NSCLC, SCLC, or BC in our findings. Thus, the true preventive effector mechanisms of β-carotene on cancers remain unknown and require further elucidation. A recent meta-analysis investigated the association between the blood levels of vitamin B6 and cancer risk (Mocellin et al., 2017). The results of that study showed preventive effects of vitamin B6 on lung and colorectal cancer risks. The lack of a significant association between vitamin B6 and BC and PC risk in that study was consistent with the results of our study. Vitamin B6 may serve as a pre-diagnostic marker of specific cancers rather than a causal factor based on the outcomes of observational studies. A recent MR analysis investigating the causal relationship between circulating B vitamins and digestive system cancers and the causal association between vitamin B6 and colorectal cancers and ECs is in line with our findings (Yuan et al., 2021). As for vitamins C and E, plausible non-causal associations exist between vitamin C and colorectal and esophageal cancer risks and between vitamin E and breast, colorectal, esophageal, lung, and PC risks (Larsson et al., 2022; Xin et al., 2022). These non-causal relationships from the published results align with the outcomes of our study. Overall, the role of exogenous antioxidants in tumor prevention warrants further exploration.

Our study had several strengths. This was the first large-scale MR analysis that systematically assessed the causal relationship between circulating antioxidants and multiple cancer risks, utilizing summary data from GWAS studies containing 193,673 solid tumor cases and controls. In addition, since short-term diet-derived antioxidant intake may not change cancer risk, actual serum concentrations of antioxidants were evaluated in the MR analysis. Moreover, the design of MR studies avoids the potential risks and harms of exposure to antioxidants in a randomized controlled trial. Nevertheless, this study also had several limitations. First, due to the lack of detailed individual information regarding sex, mental and physical conditions, and other concomitant diseases, subgroups that may show more beneficial effects of antioxidants could not be selected (Lu et al., 2022). Second, the synergistic and antagonistic interactions among antioxidants could not be evaluated; this effect may be important in realistic situations. Third, reverse-direction MR analysis between exogenous antioxidants and cancers was not performed in this study, considering the objectives of investigating the role of antioxidants in tumor prevention, the identification of non-causal relationships in forward-direction analysis, and the fact that exogenous antioxidants are supplemented mainly through dietary sources. Finally, the population cohort in this research was restricted to individuals of European ethnicity, limiting the universality of our results. Future large-power databases with more detailed individual information are warranted to verify our findings and address the limitations outlined above.

## Conclusion

In summary, this MR study provides genetic evidence of a causal association between antioxidants and multiple cancer risks in Europeans. Higher levels of circulating albumin may reduce the risk of PC and serve as a potential biomarker. Further exploration of the possible underlying mechanisms underlying these associations is essential.

## Data Availability

The original contributions presented in the study are included in the article/Supplementary Material, further inquiries can be directed to the corresponding authors.
