# Supplementary material for: Role of endogenous and exogenous antioxidants in risk of six cancers: evidence from the Mendelian randomization study
Source: Front Pharmacol. 2023 Jun 27;14:1185850. doi: 10.3389/fphar.2023.1185850 (PMC10333497; doi:10.3389/fphar.2023.1185850)
Supplement: Supplementary file 2 [file DataSheet1.DOCX]

**Role of Endogenous and Exogenous Antioxidants in Risk of Six Cancers: Evidence from the Mendelian Randomization Study**

Jiahao Zhu, Jie Lian, Xin Wang, Ren Wang, Xiangyi Pang, Benjie Xu, Xing Wang, Chenyang Li, Shengjun Ji, Haibo Lu

**Supplementary Methods and Tables**

**Supplementary Methods**

**MR-Egger method**

In addition to test and estimate causal effects (similar to IVW), MR-Egger can also test for directional pleiotropy [1]. Compared to IVW that sets the intercept term to be 0, MR-Egger adds a parameter 𝜃_0_. If the intercept term 𝜃_0_ is exactly equal to 0, then the MR-Egger estimate will be equal to the IVW estimate. Otherwise, there are pleiotropic effects independently distributed from the effect of IVs on outcomes through exposures. This means that Assumption 3 in Figure 1 will not be met. Hence, the test for whether the intercept term 𝜃_0_ is exactly equal to 0 can be referred to as the pleiotropy test [1].

**Weighted median method**

Weighted median is another MR method to calculate the causal estimate by combining data of multiple genetic IVs. The main advantage of the Weighted median is that the causal estimator is consistent even when up to 50% of the information comes from invalid IVs. The weighted median method has better finite-sample Type 1 error rates than the IVW, and is complementary to MR-Egger [2].

**Mendelian Randomization Pleiotropy Residual Sum and Outlier**

MR-PRESSO can find the outlier IVs that cause pleiotropy. MR-PRESSO can obtain an outlier corrected causal estimator [3]. Usually, no more than 50% of the genetic pleiotropy can be excluded by removing the IV outliers.

**Statistical power analysis**

We used the *F*-statistic to evaluate the strength of the IVs. The *F*-statistic can estimate the minimum detectable magnitude of the causal association in MR. In the univariable MR, the *F*-statistic and statistical power were calculated using an online tool, mRnd [4]. mRnd uses the non-centrality parameter to calculate power estimates. In addition, the fixed sample size, the heritability of exposure, and the true causal association between the exposure and the outcome were used as parameters. In the implementation, we used a 2-sided type I error rate of 0.05. In addition to the total number of individuals and proportion of cases, we provided the proportion of R^2^ variance based on SNP-Heritability listed in Table 1. We also provided the minimum detectable OR based on the IVW results. The value of 10 or higher is commonly used as the threshold of the *F*-statistic [5].

In the multivariable MR, we calculated a conditional *F-statistic* by regressing the IVs upon their corresponding exposure, conditioning on the remaining included exposures. The conditional *F-statistic* was independent of outcome because method did not use any information about the outcome.

**Heterogeneity test**

We tested the heterogeneity in the Wald-type estimators from IVs using Cochran’s Q test [6]. A *P-value* ≤ 0.10 was used as threshold to determine if there was heterogeneity.

**Reference**

[1] S. Burgess, S.G. Thompson, Interpreting findings from Mendelian randomization using the MR-Egger method, Eur J Epidemiol, 32 (2017) 377-389.

[2] J. Bowden, G. Davey Smith, P.C. Haycock, S. Burgess, Consistent Estimation in Mendelian Randomization with Some Invalid Instruments Using a Weighted Median Estimator, Genet Epidemiol, 40 (2016) 304-314.

[3] M. Verbanck, C.Y. Chen, B. Neale, R. Do, Detection of widespread horizontal pleiotropy in causal relationships inferred from Mendelian randomization between complex traits and diseases, Nat Genet, 50 (2018) 693-698.

[4] M.J. Brion, K. Shakhbazov, P.M. Visscher, Calculating statistical power in Mendelian randomization studies, Int J Epidemiol, 42 (2013) 1497-1501.

[5] G. Liu, Y. Zhao, S. Jin, Y. Hu, T. Wang, R. Tian, Z. Han, D. Xu, Q. Jiang, Circulating vitamin E levels and Alzheimer's disease: a Mendelian randomization study, Neurobiol Aging, 72 (2018) 189 e181-189 e189.

[6] Z. Han, T. Wang, R. Tian, W. Zhou, P. Wang, P. Ren, J. Zong, Y. Hu, S. Jin, Q. Jiang, BIN1 rs744373 variant shows different association with Alzheimer's disease in Caucasian and Asian populations, BMC Bioinformatics, 20 (2019) 691.

**Supplementary Tables**

Table S1-7 are available in supplementary excel files
